# Transthoracic fine-needle aspiration of the lungs, and bacterial culture with antimicrobial susceptibility testing used in diagnosing bacterial pneumonia in dogs and cats: 14 dogs and 2 cats (2009–2021)

**DOI:** 10.3389/fvets.2025.1521793

**Published:** 2025-03-12

**Authors:** Aleksandra Preibisz, Claudia Sabine Schwedes

**Affiliations:** Anicura Kleintierspezialisten Augsburg, Augsburg, Germany

**Keywords:** pulmonary FNA, transthoracic FNA, bacterial pneumonia, bacterial infection, pneumonia

## Abstract

Transthoracic fine-needle aspiration of the lungs (TFNA) combined with positive bacterial culture is used less frequently than airway lavage in diagnosing bacterial pneumonia in dogs and cats. This retrospective study evaluated TFNA results and bacterial culture findings in 14 dogs (1 dog twice) and two cats with bacterial pneumonia. Bacterial culture yielded positive results in 9/16 (56.3%) of samples. Only 1/11 (9.1%) of isolated bacterial species showed resistance to empiric treatment (amoxicillin with clavulanate). Six patients without prior antimicrobial treatment had negative bacterial cultures, despite the presence of intracellular bacteria on cytology samples. For patients with suspected bacterial pneumonia, we should consider performing both cytological examination and bacterial culture of lung aspirates, with careful interpretation of the results. Bacterial culture should be considered regardless of ongoing or previous antibiotic treatment, and even in cases where intracellular bacteria are not identified on cytology. Based on our antimicrobial susceptibility findings, first-line empirical treatment with amoxicillin with clavulanate might be an appropriate choice in most of cases regardless of the previous treatment.

## 1 Introduction

Bacterial pneumonia in dogs and cats is traditionally diagnosed through a combination of airway lavage cytology showing intracellular bacteria and positive bacterial culture results (1, 2). While bronchoalveolar lavage and transtracheal wash are considered gold standard techniques for airway sampling, transthoracic fine-needle aspiration of the lungs (TFNA) represents an alternative diagnostic approach that so far has received less attention in the literature. Previous studies have demonstrated that TFNA can effectively identify bacterial infections through cytological examination (3, 4).

TFNA offers several potential advantages over airway lavage techniques, including the ability to directly sample affected lung regions, and the option to perform the procedure without general anesthesia. However, the relationship between cytological findings and bacterial culture results from TFNA samples has not been studied extensively in clinical cases of canine or feline bacterial pneumonia.

The primary aim of this retrospective study was to evaluate the diagnostic utility of TFNA by comparing cytological findings with bacterial culture results in dogs and cats suffering from bacterial pneumonia. Additionally, we assessed the antimicrobial susceptibility patterns of isolated bacterial species to provide insights into potential antimicrobial resistance.

## 2 Materials and methods

### 2.1 Study design and inclusion criteria

Medical records of dogs and cats diagnosed with bacterial pneumonia between 2009 and 2021 at a small animal referral hospital in Germany were reviewed retrospectively. The cases were included if they met the following criteria: TFNA of the lungs was performed, and bacterial pneumonia was confirmed either by obtaining positive bacterial culture from the aspirate, or by identifying intracellular bacteria on cytology samples. Dual-view thoracic radiographs were performed for all patients. Data collected included patient signalment, prior antimicrobial treatment, clinical signs, cytological findings, results of aerobic and anaerobic bacterial culture with antimicrobial susceptibility testing, and details of antimicrobial treatment (Table 1).

**Table 1:** Clinical characteristics of the assessed dogs and cats with bacterial pneumonia, including signalment, clinical signs, prior antimicrobial treatment, cytological results, aerobic and anaerobic bacterial culture, *in vitro* bacterial susceptibility testing results and antimicrobial treatment.

Nr	Species	Sex	Age	Clinical signs	Antimicrobial treatment prior to the presentation	Intracellular bacteria	Aerobic bacterial culture	Anaerobic bacterial culture	Antimicrobial resistance	Antibiotic treatment	Outcome
1	Dog	MN	3	Nasal discharge, vomiting	No	Yes	Beta-hemolytic *Streptococcus* spp.	Not performed	Tetracycline (R), clindamycin (R)	Amoxicillin with clavulanate	Euthanasia after 2 days of treatment
							*E. coli*		Amoxicillin (R), ampicillin (R), tetracycline (R), doxycycline (R)		
2	Dog	M	5	Fever, cough	Yes, not documented	Yes	Negative	*Bacteroides pyogenes*	No information about antimicrobial resistance	Amoxicillin with clavulanate	Discharged
3	Cat	MN	2	Fever, cough	No	Yes	*E. coli*	Not performed	Cefalexin (R)	Amoxicillin with clavulanate	Euthanasia due to cavernous lung lesions and thoracic effusion after 3 weeks
4	Dog	FS	9	Respiratory distress, cough	No	Yes	*E. coli*	Not performed	Cefalexin (R)	Amoxicillin with clavulanate	Discharged
5	Dog	MN	3	Fever, cough	No	Yes	*E. coli*	Not performed	Amoxicillin (R), ampicillin (R), cefalexin (R), tetracycline (R), doxycycline (R), sulfamethoxazole with trimethoprim (R)	Amoxicillin with clavulanate	Euthanasia due to sepsis
6	Dog	MN	14	Fever, cough	Enrofloxacin	Yes	*Klebsiella pneumoniae*	Negative	Penicillin (R), amoxicillin (R), ampicillin (R), pradofloxacin (I)	Amoxicillin with clavulanate	Discharged
7	Dog	FS	8	Edema of the limbs, fever, cough	Marbofloxacin, amoxicillin with clavulanate	Yes	*Staphylococcus. intermedius*	Not performed	Penicillin (R), amoxicillin (R), ampicillin (R), tetracycline (R), doxycycline (R), pradofloxacin (I), enrofloxacin (I)	Amoxicillin with clavulanate	Discharged
8	Dog	MN	7	Weakness, cough	No	Yes	Negative	Negative	n.a.	Amoxicillin with clavulanate	Discharged
9	Dog	M	13	Respiratory distress, Cough	No	Yes	Negative	Not performed	n.a.	Amoxicillin with clavulanate	Discharged
10	Dog	F	4	Vomiting	No	Yes	Negative	Not performed	n.a.	Amoxicillin with clavulanate	Discharged
11	Dog	M	13	Vomiting, diarrhea	No	Yes	Negative	Negative	n.a.	Amoxicillin with clavulanate	Discharged
12	Dog	M	11	Dyspnea, cough	No	Yes	Negative	Negative	n.a.	Amoxicillin with clavulanate	Discharged
13	Dog	MN	13	Cough, respiratory distress, vomiting	Enrofloxacin	Yes	Negative	Not performed	n.a.	Amoxicillin with clavulanate	Discharged
14	Cat	M	4 weeks	Respiratory distress	Amoxicillin with clavulanate	Yes	Not performed	Not performed	n.a.	Amoxicillin with clavulanate	Discharged
15	Dog	FS	13	Vomiting, diarrhea	No	No	*Klebsiella pneumoniae*	Not performed	Penicillin (R), amoxicillin (R), ampicillin (R)	Amoxicillin with clavulanate	Discharged
16A	Dog	F	6	Regurgitation	No	Yes	Negative	Negative	n.a.	Amoxicillin with clavulanate	Discharged
16B	Dog	F	6	Cough	Amoxicillin with clavulanate	Yes	*E. coli*	Negative	Penicillin (R), amoxicillin (R), ampicillin (R), pradofloxacin (I)	Enrofloxacin	Discharged
							*Enterobacter cloacae*		Penicillin (R), amoxicillin (R), ampicillin (R), amoxicillin with clavulanate (R), cefalexin (R), tetracycline (R), doxycycline (R)		

F, female intact; FS, female spayed; M, male intact; MN, male neutered; R, resistant, I, intermediate; R, resistant; n.a., not applicable.

### 2.2 Transthoracic fine-needle aspiration procedure

TFNA was performed within 6 h of admission. Lesion localization was guided by thoracic radiographs and ultrasound examination. When visible on ultrasound, consolidated lung regions appearing as hypoechoic areas (“shred sign”) adjacent to the pleural surface were targeted. In cases where peripheral lesions were not ultrasonographically visible, TFNA was performed based on radiographic findings. The applied method of the TFNA (blindly performed or ultrasound-guided) was not recorded in all cases. 25G hypodermic needle was used for all aspiration procedures Flow-by oxygen supplementation was provided during the procedure for all the patients. Sedation with butorphanol (Dolorex® 10 mg/mL; MSD; 0.2–0.3 mg/kg IM or IV) was administered only to uncooperative patients. Samples for cytology and bacterial culture were collected from the same anatomical site.

### 2.3 Sample processing and analysis

Cytological samples were air-dried, stained with Diff-Quick (LABOR+TECHNIK Eberhard Lehmann GmbH, Germany), and evaluated by a board-certified small animal internal medicine specialist. A diagnosis of bacterial pneumonia was made when samples showed increased numbers of neutrophils with intracellular bacteria. Cases with increased neutrophils, but no visible bacteria were classified as purulent inflammation.

### 2.4 Bacterial culture and antimicrobial susceptibility testing

Samples with increased neutrophils were submitted to the external laboratory (IDEXX Laboratories, Germany) for bacterial culture within 24 h of collection. Both aerobic and anaerobic cultures were performed when sample volume was sufficient. In only 7/17 cases (41%), the TFNA yielded adequate volume for both aerobic and anaerobic bacterial culture procedures. Antimicrobial susceptibility testing was conducted on all bacterial isolates. The exact MIC breakpoints for each testing usually were not provided by the laboratory.

### 2.5 Post-procedure monitoring

All the animals were monitored in the intensive care unit for at least 24 h following TFNA to assess for potential complications. Clinical outcome (discharge or euthanasia) was recorded for all patients.

## 3 Results

### 3.1 Clinical characteristics of dogs and cats under evaluation

The study included 16 animals (14 dogs and 2 cats), with one dog (case nr 16) undergoing TFNA twice, due to recurrent aspiration pneumonia associated with megaesophagus, resulting in 17 samples. The median age was 8.5 years (range: 3–14 years) for the dogs, whereas the two cats were 4 weeks and 2 years old, respectively.

6/17 of samples (35.3%) were obtained from patients that had received prior antimicrobial treatment, including enrofloxacin (*n* = 2), marbofloxacin with amoxicillin with clavulanate (*n* = 1), amoxicillin with clavulanate (*n* = 2) and unknown antimicrobial drug (*n* = 1). The duration of previous antimicrobial treatment was not documented. The most common clinical presentations included respiratory signs (cough, respiratory distress), gastrointestinal signs (vomiting, regurgitation), and systemic signs (fever). In three dogs aspiration pneumonia associated with megaesophagus (*n* = 1), surgical arytenoid lateralization (*n* = 1), and seizures (*n* = 1) were confirmed. Most patients tolerated TFNA without sedation; only one dog required sedation with butorphanol (0.2 mg/kg IM).

### 3.2 Cytological examination and bacterial culture results

Cytological examination revealed intracellular bacteria in 16/17 samples (94.1%). Bacterial culture was performed on 16/17 samples (94.1%), with both aerobic and anaerobic cultures conducted in 8/16 cases (50%). Only the sample obtained from 4-week-old kitten was insufficient for performing bacterial culture. 9/16 cultured samples (56.3%) yielded positive results, including the presence of *Escherichia coli* (*n* = 5), *Klebsiella pneumoniae* (*n* = 2), *Staphylococcus intermedius* (*n* = 1), *β*-hemolytic *Streptococcus* spp. (*n* = 1), *Enterobacter cloacae* (*n* = 1), and *Bacteroides pyogenes* (*n* = 1). 2/9 (22.2%) of positive cultures showed polymicrobial infection. 6/17 of the samples (35.3%) were obtained from patients that had received prior antimicrobial treatment. In 4 of these 6 patients, their bacterial culture results returned positive although they received antimicrobial therapy before the TFNA. The remaining 2 pre-treated patients had either no bacterial culture performed or received a negative result. 6/11 (54.5%) patients without prior antimicrobial treatment had negative bacterial cultures, despite the intracellular bacteria in cytology. Additionally, one sample resulted in a positive culture (*Klebsiella pneumoniae*) without cytological evidence of bacteria.

### 3.3 Antimicrobial susceptibility

Antimicrobial susceptibility testing revealed widespread resistance to pure *β*-lactams (penicillin, amoxicillin, ampicillin) and tetracyclines, as well as frequent resistance to cefalexin. Some isolates showed intermediate susceptibility to fluoroquinolones (pradofloxacin, enrofloxacin). Only 1/11 (9.1%) of the isolates showed resistance to amoxicillin with clavulanate.

### 3.4 Outcome

Among the 16 animals, 14 (87.5%) were discharged (12 dogs and both cats). Two dogs were euthanized: one shortly after sampling due to severe pneumonia and sepsis, and another one after 2 days of treatment. The 2-year-old cat was readmitted 3 weeks post-discharge with thoracic effusion and cavitary lung lesions (suspected pulmonary abscess). No immediate complications were reported following TFNA procedures in any patient.

## 4 Discussion

This study demonstrated that TFNA of the lungs is a valuable diagnostic tool for bacterial pneumonia in dogs and cats. In patients with negative bacterial cultures, we frequently observed intracellular bacteria on TFNA cytology. Patients that received antimicrobial therapy before the TFNA, had often positive bacterial culture results.

Bacterial species (*Escherichia coli, Staphylococcus intermedius, Streptococcus* spp.) isolated in our study are pathogens commonly isolated from dogs and cats with bacterial pneumonia (2, 5–11). *Klebsiella pneumoniae*, isolated in two of our patients, is likewise commonly isolated (5, 6, 8–11). Isolation of obligate anaerobes like *Bacteroides pyogenes* in our study highlights the importance of anaerobic culture. One study reported that from the samples obtained from dogs with suspected lower respiratory tract disease, 21.6% were obligate anaerobes, with Bacteroides species being among the most common anaerobic isolates (9). However, since only half of our samples underwent anaerobic culture, we may have underestimated the true prevalence of anaerobic infections in our patients. Our study did not isolate *Bordetella bronchiseptica*, which differs from findings in other studies (5–7, 9–12). The reason for this could be performing TFNA in patients with bacterial pneumonia instead of bronchoalveolar lavage or tracheal wash reported in the literature.

We found polymicrobial infections in 2/17 (11.8%) samples, in dogs with either confirmed or suspected aspiration pneumonia. Polymicrobial infections were reported frequently in other studies (6, 7, 9–12) but could not be linked solely to aspiration pneumonia. While six of our patients presented with vomiting or regurgitation, aspiration could be confirmed in only three cases. Interestingly, four of the remaining cases yielded single bacterial isolates, which may have made aspiration pneumonia less likely, though did not exclude it at the same time.

The discrepancies we observed between cytology and bacterial culture results (negative cultures with positive cytology and vice versa) have been reported previously, though are relatively uncommon (2, 6) and thus should be interpreted carefully due to possible contamination. However, the study in experimentally infected guinea pigs showed that bacterial culture could identify the infectious agent in only 45.5% of confirmed cases, suggesting that cytological examination positive for bacteria should not be dismissed as sample contamination when bacterial culture is negative (13).

The discrepancy between cytology and bacterial culture could be caused by several factors. Firstly, bacterial growth could be compromised by prior antimicrobial treatment, or the causative agent could be difficult to culture. Secondly, separate aspirations for cytology and culture could sample different areas of the lungs. Thirdly, culture cannot differentiate between intra-and extracellular bacteria, whereas our cytological criterion for bacterial pneumonia was the presence of intracellular bacteria. Lastly, although samples were taken aseptically, there was always some risk of contamination with opportunistic pathogens.

The 35.3% incidence of prior antimicrobial treatment in our population resulted in relatively low bacterial resistance (9.1%), compared to another study reporting 57.4% resistance to previously administered antimicrobials (5). The substantial discrepancy in antimicrobial resistance could be caused by different bacterial susceptibility patterns in North America and Europe. This discrepancy could result from the differences in antibiotic use in these geographic regions. However, studies in human medicine did not confirm increased antimicrobial resistance due to increased antibiotic use (14). We observed minimal bacterial resistance to amoxicillin-clavulanate. Although the study (15) showed a good clinical response to this antimicrobial drug in dogs with aspiration pneumonia, it should be noted that no bacterial culture was performed, so antimicrobial susceptibility was not evaluated. It is important to emphasize that our sample of 11 isolates may be insufficient to formulate general recommendations for an empirical treatment plan.

Study limitations include its retrospective nature with incomplete documentation of prior treatment, small sample size, potentially introducing bias, and inconsistent use of anaerobic culture potentially underestimating anaerobic infections.

While, as demonstrated in several studies (5–12), bronchoalveolar lavage and transtracheal wash are considered gold standard techniques for airway sampling in respiratory infections, TFNA may offer a quick and low-cost diagnostic procedure in unstable patients. In our study, TFNA provided diagnostic samples adequate for both cytology and culture in most of the cases. The only case where bacterial culture was not performed was a 4-week old kitten, with sample size being insufficient for both procedures. What is important, our study showed that TFNA can yield clinically relevant culture and susceptibility results to guide antimicrobial therapy when airway lavage may not be available. However, larger comparative studies are still needed to fully establish the diagnostic utility of these different sampling techniques.

## 5 Conclusion

This study demonstrated that TFNA is an effective diagnostic tool for bacterial pneumonia in dogs and cats. For patients with suspected bacterial pneumonia, we should consider performing both cytological examination and bacterial culture of lung aspirates, with careful interpretation of the results. Bacterial culture should be considered regardless of ongoing or previous antibiotic treatment, and even in cases where intracellular bacteria are not identified on cytology. Based on our antimicrobial susceptibility findings, first-line empirical treatment with amoxicillin with clavulanate might be an appropriate choice in most of cases regardless of the previous treatment.

## Data Availability

The original contributions presented in the study are included in the article/supplementary material, further inquiries can be directed to the corresponding author.
